# Optic Nerve Head Change in Non-Arteritic Anterior Ischemic Optic Neuropathy and Its Influence on Visual Outcome

**DOI:** 10.1371/journal.pone.0037499

**Published:** 2012-05-21

**Authors:** Jost B. Jonas, Sohan Singh Hayreh, Yong Tao, Konstantinos I. Papastathopoulos, Florian Rensch

**Affiliations:** 1 Department of Ophthalmology, Medical Faculty Mannheim of the Ruprecht-Karls-University Heidelberg, Mannheim, Germany; 2 Departments of Ophthalmology and Visual Sciences, College of Medicine, University of Iowa, Iowa City, Iowa, United States of America; 3 Department of Ophthalmology, People's Hospital, Peking University, Beijing, China; Center of Ophtalmology, Germany

## Abstract

**Purpose:**

To evaluate changes in cup/disc (C/D) diameter ratios and parapapillary atrophy in patients with non-arteritic anterior ischemic optic neuropathy (NA-AION), using morphometric methods.

**Methods:**

The clinical non-interventional study included 157 patients with unilateral or bilateral NA-AION. Optic disc photographs taken from both eyes at the end of follow-up were morphometrically examined.

**Results:**

Follow-up was 86.3±70.3 months. Horizontal and vertical disc diameters (*P* = 0.30;*P* = 0.61, respectively), horizontal and vertical C/D ratios (*P* = 0.47;*P* = 0.19,resp.), and size of alpha zone and beta zone of parapapillary atrophy (*P* = 0.27;*P* = 0.32,resp.) did not differ significantly between affected eyes and contralateral normal eyes in patients with unilateral NA-AION. Similarly, horizontal and vertical disc diameters, horizontal and vertical C/D ratios, and size of alpha zone and beta zone did not vary significantly (all *P*>0.05) between the unaffected eyes of patients with unilateral NA-AION and the eyes of patients with bilateral NA-AION. Optic disc diameters, C/D ratios, size of alpha zone or beta zone of parapapillary atrophy were not significantly associated with final visual outcome in the eyes affected with NA-AION (all *P*>0.20) nor with the difference in final visual acuity between affected eyes and unaffected eyes in patients with unilateral NA-AION (all *P*>0.25).

**Conclusions:**

NA-AION did not affect C/D ratios nor alpha zone and beta zone of parapapillary atrophy. Optic disc size was not related to the final visual acuity outcome in NA-AION.

## Introduction

Non-arteritic anterior ischemic optic neuropathy (NA-AION) is an acute ischemic disorder of the optic nerve head [1], occurring with an incidence rate of about 2 to 10 per 100,000 subjects [2]–[5]. Although the association of NA-AION with absent or a small cup/disc ratio (C/D) has been reported by several studies since 1974 [6]–[20], little information on the following aspects about NA-AION has been gathered so far:

1. Whether within the groups of patients with NA-AION, eyes with the smaller optic disc have a higher risk to become affected?
2. Whether there are differences between affected eyes and unaffected eyes of the same individuals in the size of alpha zone and beta zone of parapapillary atrophy at the end of the follow-up [21], [22], in addition to the question whether affected eyes and unaffected eyes of the same individuals differ in size and shape of the neuroretinal rim at the end of the follow-up
3. Whether final visual outcome in the affected NA-AION eyes is associated with the optic disc diameter, cup/disc diameter (C/D) ratio, or size of alpha zone or beta zone of parapapillary atrophy.

The objective of the present study was, therefore, to investigate the above aspects of NA-AION eyes by measuring the optic nerve head by a morphometric method in a larger number of patients with: (i) unilateral NA-AION and (ii) bilateral NA-AION, several months after the acute onset of the disorder, when the optic disc edema had resolved.

## Methods

### Ethics Statement

The Institutional Review Board of the College of Medicine, University of Iowa, approved the study protocol, and all patients agreed upon inclusion into this observational study. At the time when the study was designed, a written consent of patients undergoing a routine non-invasive diagnostic evaluation and follow-up was not necessary. A written consent was required for any invasive procedure none of which was performed in the present observational study.

The prospective clinical non-observational study included a cohort of patients with NA-AION, who were prospectively examined and diagnosed by the same clinician (SSH) in the Ocular Vascular Clinic at the University of Iowa Hospitals and Clinics, as a part of a National Institute of Health funded (RO1) prospective study. The study population was divided into a subgroup of patients with unilateral NA-AION and a second subgroup of patients with bilateral NA-AION. In patients with bilateral NA-AION, the optic neuropathy involved one eye before the second eye. The patients with bilateral NA-AION entered the study when the second eyes were acutely affected. The ratio between the unilateral and bilateral NA-AION in this study does not reflect the actual prevalence seen [1].

#### Criteria required for diagnosis of NA-AION and inclusion in this study

These included:

1. A history of sudden visual loss, usually discovered in the morning; and this was not due to any ocular, systemic or neurological diseases.
2. Optic disc edema at onset must have been documented in the Ocular Vascular Clinic or by another ophthalmologist.
3. Spontaneous resolution of optic disc edema was observed.
4. The eye had optic disc-related visual field defects.
5. . Satisfactory stereoscopic photographs of both optic discs had to be available at baseline and the end of follow-up.

#### Exclusion criteria

All patients with any ocular, systemic or neurological diseases, that might influence or explain the patient's visual loss, were excluded. NA-AION patients with only background diabetic retinopathy were included, but those who had active neovascularization, vitreous hemorrhages, traction detachment or other complications influencing the visual acuity or fields were excluded. Patients who had a diagnosis of glaucoma were excluded.

At the first visit to our clinic, a detailed ophthalmic and medical history was obtained from all these patients (by SSH). A complete ophthalmic evaluation was performed at that time (by SSH) for each patient; this included Snellen visual acuity, visual fields plotted with a Goldmann perimeter, relative afferent pupillary defect, intraocular pressure measurement, slit lamp examination of the anterior segment, direct and indirect ophthalmoscopy, stereoscopic color fundus photography, and in acute cases stereoscopic fundus fluorescein angiography. Stereoscopic color fundus photographs of both eyes were taken at the initial visit and later on. At the same time, a detailed systemic evaluation was performed by a cardiologist, internist or the patient's local physician. If giant cell arteritis was suspected, a temporal artery biopsy was performed. The erythrocyte sedimentation rate and the serum concentration of the C-reactive protein were within the age-related limits for all patients included into the study. None of the patients included into the study complained about symptoms typical for giant cell arteritis, jaw claudication, anorexia and unintended weight loss in the months preceding the onset of the acute vision loss, nor had positive temporal artery biopsy for giant cell arteritis. If indicated, patients had carotid duplex scans, carotid angiograms and/or echocardiography. The study design has been described in detail previously [17], [23].

The optic disc photographs of both eyes of the patients in this study were digitized and we assessed and measured the horizontal and vertical optic cup and optic disc diameters and the presence and maximal width of alpha zone and beta zone of parapapillary atrophy. The optic disc was defined as all area within the peripapillary scleral [21], [22]. The optic cup was defined on the basis of contour and not of color. Parapapillary atrophy was differentiated into alpha zone and beta zone. Alpha zone was defined as peripheral zone with irregular hyperpigmentation and hypopigmentation, and beta zone was characterized by visible large choroidal vessels and visible sclera [21], [22]. If beta zone was present, it was adjacent to the peripapillary scleral ring and interposed between this ring and alpha zone. All optic disc structures were measured in relative size units as given by the planimetric computerized measurement program. Masking of the examiners was not possible since the diagnosis could be detected on the appearance (i.e. of the optic disc), which was to be measured. The optic disc images taken in eyes with unilateral NA-AION as well as in the fellow normal eyes were examined at the end of the follow-up period. This was because at the end of the follow-up, the optic disc edema had completely resolved, so that the border of the optic disc (i.e. the inner margin of the peripapillary scleral ring) and the border between the optic cup and neuroretinal rim could be clearly visualized. In a similar manner in patients with bilateral NA-AION, stereoscopic photographs of both optic discs taken at the end of follow-up were morphometrically examined.

The statistical analysis was performed using a commercially available statistical software package (SPSS for Windows, version 19.0, SPSS, Chicago, IL). The data were presented as mean ± standard deviation (SD). Only measurements of optic nerve head images obtained at the end of the follow-up were used for the statistical analysis. For comparison between the affected eyes and the contralateral unaffected eyes in the patients with unilateral NA-AION, the non-parametric Wilcoxon test for paired samples was applied. For the comparison of optic disc measurements between patients with unilateral NA-AION and patients with bilateral NA-AION, the non-parametric Mann-Whitney test for un-paired samples was taken. All *P*-values were 2-sided and were considered statistically significant when the values were less than 0.05.

## Results

The study comprised 157 patients (38.9% women) with a mean age of 58.5±12.1 years (median: 59.8 years). The mean follow-up was 86.3±70.3 months (median: 74.1 months). Of the 157 patients included in this study, 70 (32 in the right eye and 38 in the left) had unilateral NA-AION, and 87 bilateral. These two groups did not vary significantly in age (*P* = 0.38) nor in gender (*P* = 0.26).

### Optic Disc Diameters

In patients with unilateral NA-AION (n = 70), comparing the affected eyes with the unaffected contralateral eyes revealed that the horizontal disc diameter (*P* = 0.30) and the vertical disc diameter (*P* = 0.61) did not differ significantly (Table 1). Comparing the optic disc diameters of the unaffected eyes of the patients with unilateral NA-AION (n = 70) with the optic disc diameters of the patients with bilateral NA-AION (n = 87) showed that the horizontal optic disc diameter did not vary between the unaffected eyes in the unilateral NA-AION group versus both eyes in the bilateral NA-AION group (unaffected eyes versus for right eyes of bilaterally affected patients: *P* = 0.06; and versus left eyes of bilaterally affected patients: *P* = 0.11). In a similar manner, the vertical optic disc diameter did not vary between the unaffected eyes in the unilateral NA-AION group versus both eyes in the bilateral NA-AION group (unaffected eyes versus right eyes of bilaterally affected patients: *P* = 0.43; and versus left eye of bilaterally affected patients: *P* = 0.26) (Table 1).

**Table 1:** Measurements (Mean ± Standard Deviation) of Optic Disc Parameters in Patients with unilateral or bilateral non-arteritic anterior ischemic optic neuropathy (NA-AION).

	Unilateral NA-AION			Bilateral NA-AION	
	Affected Eye	Unaffected Eye	*P*-Val.	Right Eyes	Left Eyes
**Optic Disc Diameter**					
Horizontal	4.54±0.49	4.61±0.48	0.30	4.49±0.44 (P* = 0.11)	4.66±0.53 (P* = 0.06)
Vertical	4.71±0.57	4.84±0.52	0.61	4.74±0.63 (P* = 0.26)	4.81±0.54 (P* = 0.43)
**Cup/Disc Diameter Ratio**					
Horizontal	0.19±0.14	0.20±0.11	0.47	0.18±0.12 (P* = 0.33)	0.22±0.15 (P* = 0.07)
Vertical	0.21±0.15	0.20±0.12	0.19	0.18±0.12 (P* = 0.22)	0.24±0.16 (P* = 0.06)

*P*-Val.: Statistical significance of the difference between the affected eyes and the unaffected eyes of patients with unilateral NA-AION.

P*: Statistical significance of the difference between the eyes of patients of bilateral NA-AION and the unaffected eyes of patient with unilateral NA-AION.

### Cup/Disc Diameter Ratios

In patients with unilateral NA-AION (n = 70), a comparison of the affected eyes with the unaffected contralateral eyes revealed that the horizontal C/D ratio (*P* = 0.47) and the vertical C/D ratio (*P* = 0.19) did not differ significantly between both eyes (Table 1). Comparing the C/D ratios of the unaffected eyes of the patients with unilateral NA-AION (n = 70) with the C/D ratios of the patients with bilateral NA-AION (n = 87) showed that the horizontal C/D ratio did not differ between the unaffected eyes in the unilateral NA-AION group versus either eye in the bilateral NA-AION group (unaffected eyes versus right eyes of bilaterally affected patients: *P* = 0.07; and versus left eyes of bilaterally affected patients: *P* = 0.33). In a similar manner, the vertical C/D ratio did not vary between the unaffected eyes in the unilateral NA-AION group versus either eye in the bilateral NA-AION group (unaffected eyes versus right eye of bilaterally affected patients: *P* = 0.06; and versus left eyes of bilaterally affected patients: *P* = 0.22) (Table 1). The C/D ratios and the disc size did not differ between the affected eyes and the unaffected eyes of patients with unilateral NA-AION; similarly, the size and shape of the neuroretinal rim and optic cup did not differ between the two eyes.

### Parapapillary atrophy

In patients with unilateral NA-AION, comparison of the affected eyes with the unaffected contralateral eyes revealed that the greatest diameter of alpha zone (0.61±0.44 units versus 0.75±0.38 units; *P* = 0.27) and beta zone (0.23±0.49 units versus 0.19±0.51 units; *P* = 0.32) did not differ significantly between the two eyes. Comparing the greatest diameters of alpha zone and beta zone of the unaffected eyes of the patients with unilateral NA-AION with the greatest diameters of alpha zone and beta zone of the patients with bilateral NA-AION showed that the greatest diameters of alpha zone did not vary between the unaffected eyes in the unilateral NA-AION group versus either eye in the bilateral NA-AION group (*P* = 0.20 and *P* = 0.66, respectively). Likewise, the greatest diameter of beta zone did not vary between the unaffected eyes in the unilateral NA-AION group versus either eye in the bilateral NA-AION group (*P* = 0.30 and *P* = 0.45, respectively).

### Final Visual Acuity

Final visual acuity in the affected right eyes was worse than 20/200 in 11.7% of the patients, and worse than 20/40 in 35.1% of the patients. Final visual acuity in the affected left eyes was worse than 20/200 in 10.5% of the patients, and worse than 20/40 in 35.5% of the patients. In patients with unilateral NA-AION, final visual acuity (logMAR) of the affected eye was not significantly associated with the horizontal or the vertical optic disc diameter, the horizontal or vertical C/D ratio, and nor the greatest diameter of alpha zone and beta zone of parapapillary atrophy in the eyes affected with NA-AION (*P* = 0.54, *P* = 0.65, *P* = 0.76, *P* = 0.71, *P* = 0.75, and *P* = 0.21, respectively) nor with the corresponding parameters of the unaffected eyes (*P* = 0.70, *P* = 0.80, *P* = 0.23, *P* = 0.11, *P* = 0.18, and *P* = 0.35, respectively).

Assuming that visual acuity did not differ significantly between both eyes of the same individual before the onset of NA-AION, we calculated the loss in visual acuity as the difference in visual acuity between the unaffected eye and the affected eye for the patients with unilateral NA-AION. It revealed that visual acuity loss was not significantly associated with the horizontal or the vertical optic disc diameter, the horizontal or vertical C/D ratio, the greatest diameter of alpha zone and beta zone of parapapillary atrophy in the eyes affected with NA-AION (*P* = 0.75, *P* = 0.73, *P* = 0.49, *P* = 0.98, *P* = 0.82, and *P* = 0.26, respectively) nor with the corresponding parameters of the contralateral eyes unaffected by NA-AION (*P* = 0.30, *P* = 0.33, *P* = 0.95, *P* = 0.68, *P* = 0.93, and *P* = 0.24, respectively).

## Discussion

Our study was performed with the objective of addressing questions in NA-AION on which there is so far little information. Following are the questions and the answers provided by our study results.

### Within the groups of patients with NA-AION, do eyes with the smaller optic disc have a higher risk of being affected?

NA-AION did occur preferentially in eyes with small optic nerve heads; but there was no statistically significant difference in optic disc size in the two eyes of patients with unilateral NA-AION, nor between patients with unilateral NA-AION and bilateral NA-AION. Saito and colleagues also found no significant difference in disc area between NA-AION affected eyes and the unaffected fellow eyes [20].

### Is there a difference between affected eyes and unaffected eyes of the same individuals in the size of alpha zone and beta zone of parapapillary atrophy at the end of the follow-up?

The alpha zone and beta zone of parapapillary atrophy did not differ between the eyes affected by NA-AION and the contralateral eyes, or between the unaffected eyes of the patients with unilateral NA-AION and the affected eyes of the patients with bilateral NA-AION. This supports a previous smaller study [13]. It shows a difference between eyes with glaucomatous optic neuropathy and NA-AION with optic nerve damage: while in glaucoma, parapapillary atrophy enlarges and shows a spatial relationship with the loss of neuroretinal rim inside of the optic disc and with the longest distance to the central retinal vessel trunk [24], parapapillary atrophy in NA-AION does not change markedly. This gives another criterion to differentiate between glaucoma and NA-AION. Interestingly, arteritic AION, like NA-AION, is also not associated with an enlargement of parapapillary atrophy, so that the loss of rim and deepening of the optic cup are the main morphological differences between the two types of AION [25]. This reflects differences in the pathogeneses of ischemic optic neuropathies (acute ischemic disorders) and glaucomatous optic neuropathies (chronic ischemic disorders) [26]–[29].

### In unilateral NA-AION, is there a difference in size and shape of the neuroretinal rim at the end of the follow-up?

Our study showed no difference in optic disc cup and C/D diameter between the affected eyes and their contralateral unaffected eyes. This confirms previous investigations [6]–[20]. It corresponds with the finding that the C/D ratios, as surrogates of the optic disc size [30], were markedly lower in our study population (mean horizontal C/D ratios: 0.20±0.13; mean vertical C/D ratio: 0.20±0.14) than in the normal eyes of the Rotterdam Study (mean horizontal C/D ratio: 0.40±0.14; vertical C/D ratio: 0.49±0.14) [31]. It agrees with the already reported association between of NA-AION and absent or small optic disc cup [6]–[20]. The results in particular confirm a previous investigation by Danesh-Meyer and colleagues [15], who applied confocal laser scanning laser tomography to quantify the morphological appearance of the optic nerve morphology in 23 patients after NA-AION. Images were taken of both affected and unaffected eyes, with the latter serving as controls. Danesh-Meyer and coworkers found that the rim area was 6% smaller (*P* = 0.13) in the affected eyes than in the fellow eyes, and the authors concluded that the optic cup after an event of NA-AION did not enlarge.

Since the C/D ratio did not differ between the affected eyes and the contralateral unaffected eyes at the end of follow-up in our study, it suggests that size and shape of the neuroretinal rim did not change due to NA-AION. This is in contrast to the loss of neuroretinal rim and widening and deepening of the optic cup observed in eyes after arteritic anterior ischemic optic neuropathy [25], [32]–[35], and in eyes with any type of glaucoma. It is not clear why the ischemic event in the vast majority of NA-AION eyes is not associated with a loss in neuroretinal rim, although optic nerve fibers are lost, as in glaucoma and as in arteritic anterior ischemic optic neuropathy. The finding puts NA-AION into the same group of non-glaucomatous optic neuropathies such optic nerve damage due to optic nerve compression or transection, or central retinal artery occlusion, in which the size and shape of the neuroretinal and optic cup do not markedly change in spite of extensive loss of nerve fibers [36]. It is possible that in these eyes there is associated secondary gliosis which replaces the optic nerve fiber loss.

### Is the final visual acuity outcome in the affected NA-AION eyes associated with the optic disc diameters, C/D ratios, or size of alpha or beta zone of parapapillary atrophy?

The study showed that not to be the case.

Our study agrees with previous investigations applying optical coherence tomography as technique to examine the optic nerve head. In a study by Suh and colleagues, the ratio of neuroretinal rim to retinal nerve fiber layer thickness showed significant differences between eyes with NA-AION and eyes with open-angle glaucoma suggesting that the neuroretinal rim was mostly preserved in the eyes with NA-AION [37]. Correspondingly, our study showed that the cup/disc ratio (and thus indirectly the neuroretinal rim) did not differ markedly between the eyes affected by NA-AION and the contralateral unaffected eyes. In a similar manner in a study by Chan and coworkers, patients with NA-AION had smaller optic cups and cup/disc area ratios in both eyes compared with controls [19]. In an investigation by Contreras and coworkers, patients with NA-AION also had lower cup/disc ratios than a normal population [38]. Interestingly, Contreras and colleagues did not find a significant difference in optic disc size between patients with NAION and the control subjects.

### Potential limitations of our study

Some may consider that our study has the following limitations. Following are our responses to that.

First, the magnification of the optic nerve head images by the optic media and the fundus camera was not corrected. Since, however, parameters such as the horizontal and vertical C/D ratios do not depend on the magnification of the optic disc photograph and since most of the findings reported in our study were based on an inter-eye comparison in individuals, the missing correction of the optic disc structure measurements should not have appreciably influenced the results and conclusions of the study.

Second, modern imaging techniques such as confocal laser scanning tomography of the optic nerve head or optical coherence tomography were not available when the patients were primarily examined in our study. These techniques can measure the three-dimensional topography of the optic nerve head and may thus have an advantage over the two-dimensional assessment of optic disc photographs. However, a study by Danesh-Meyer e al. [15], using the modern techniques, proved what our study showed.

Third, the optic nerve head measurements were taken on photographs performed after the development of NA-AION and resolution of optic disc edema. Therefore, we had to use the fellow normal eye as surrogate for the pre-NA-AION state of the optic disc in the involved eye. It would evidently have been better if photographs had been available from before the development of NA-AION but that is obviously not possible, since the patients come for consultation only when they lose vision, so this goal was not achievable. The post hoc analysis of photographs after resolution of the optic disc swelling at the end of the follow-up may therefore, have been an acceptable compromise.

Fourth, it would have been interesting not only to compare the C/D ratios as surrogate for the disc size, but additionally to compare the disc diameters between the participants of our study and the participants of population-based studies, such as the Rotterdam Study [31]. This comparison was, however, not possible since the correction of the magnification of fundus photographs varied between the studies and thus would introduce a bias.

Fifth, some may consider that the follow-up of the patients may not have been long enough. A previous study of 380 consecutive eyes with NA-AION showed, however, that optic disc edema usually resolved spontaneously in about 8 weeks [39], and after that the optic disc remained stable. A follow-up of 2 months as used in our study as inclusion criterion therefore appeared to have been adequate to provide valid information.

Sixth, the follow-up may not have been sufficient to obtain reliable information about the final visual acuity. A previous study [23] on the natural history of visual outcome in 386 eyes with NA-AION showed that 6 months after onset of NA-AION, there was no change in visual acuity and visual fields. That was also shown by the Ischemic Optic Neuropathy Decompression Trial Research Group [40]. In view of that, NA-AION patients with a minimum follow-up of 6 months for visual acuity may therefore have provided all the information required in the present study. In this study the mean follow-up was 86.3±70.3 months (median: 74.1 months) and the patients within the two subgroups were followed for longer than 6 months.

Seventh, the study design would have improved by including a normal control group. The data of the study groups of our investigation were, however, compared with the normative data of the population-based Rotterdam Study so that the data of a normal control group were indirectly included into the present study.

Eight, the unaffected eyes of the patients with unilateral NA-AION as compared to normal eyes of normal subjects have an increased risk to get affected by NA-AION sometime later on than the end of follow-up in this study [1]. The unaffected eyes of the patients with unilateral NA-AION as compared to normal eyes of normal subjects have no cup or only a small cup [6]–[20]. Therefore, using parameters of the unaffected eyes of the patients with unilateral NA-AION as a control was logical for the involved eyes. That helped, to explore the optic disc morphology after NA-AION.

Ninth, when we calculated the NA-AION induced loss in visual acuity as the difference in visual acuity between the unaffected eye and the affected eye, we assumed that the visual acuity did not differ significantly between both eyes of the same individual before the onset of NA-AION, i.e. it was normal in both eyes unless patient had noted any difference, and that disqualified the patient for this study.

### Conclusion

NA-AION did not affect C/D ratios, so that the size and shape of the neuroretinal rim and optic cup in this study were not changed by NA-AION. The unchanged size and shape of the neuroretinal rim differentiated NA-AION from arteritic AION and glaucomatous optic neuropathy. Neither alpha zone nor beta zone of parapapillary atrophy was affected by NA-AION, giving another differential criterion between NA-AION and glaucoma. Within the groups of patients with NA-AION, disc size was not related with the occurrence of NA-AION or final visual outcome.
